# Experimental evidence reveals the *UCP1* genotype changes the oxygen consumption attributed to non-shivering thermogenesis in humans

**DOI:** 10.1038/s41598-017-05766-3

**Published:** 2017-07-17

**Authors:** Takayuki Nishimura, Takafumi Katsumura, Midori Motoi, Hiroki Oota, Shigeki Watanuki

**Affiliations:** 1 Department of Biomedical Sciences, Nagasaki University, 1-12-4 Sakamoto, Nagasaki, 852-8523 Japan; 2 Department of Anatomy, Kitasato University School of Medicine, 1-15-1 Kitasato, Minami-ku, Sagamihara, Kanagawa 252-0374 Japan; 3 Deparatment of Human Science, Kyushu University, 4-9-1 Shiobaru, Minami-ku, Fukuoka 815-8540 Japan

## Abstract

Humans have spread out all over the world adapting to many different cold environments. Recent worldwide genome analyses and animal experiments have reported dozens of genes associated with cold adaptation. The uncoupling protein 1 (*UCP1*) gene enhances thermogenesis reaction in a physiological process by blocking ATP (adenosine triphosphate) synthesis on a mitochondrial membrane in brown adipose tissues. To our knowledge, no previous studies have shown an association between variants of the *UCP1* gene and physiological phenotypes concerning non-shivering thermogenesis (NST) under the condition of low temperature in humans. We showed that the degree of NST for healthy subjects in an artificial climate chamber is significantly different among *UCP1* genotypes. Defining the haplotypes covering the *UCP1* region (39.4 kb), we found that the frequency of the haplotype with the highest NST was significantly correlated with latitudes and ambient temperature. Thus, the data in this study provide the first evidence that the *UCP1* genotype alters the efficiency of NST in humans, and likely supports the hypothesis that the *UCP1* gene has been related to cold adaptation in human evolutionary history.

## Introduction

Cold adaptation in humans is one of the key factors for their great success in the out of Africa migration^1^. Not only cultural adaptation (using fire, clothes, shelter, and so on) but also physiological adaptation (thermogenesis that maintains thermal homeostasis in body temperature)^2^ must have been necessary for the expansion to the Eurasian continent. Mammals have two systems of thermogenesis: one is shivering thermogenesis (ST), and the other is nonshivering thermogenesis (NST). In ST, shivering of skeletal muscles generates heat, while in NST, the inhibition of mitochondrial adenosine triphosphate (ATP) synthesis in brown adipose tissue (BAT) generates heat^3^. Therefore, ST depends on an individual’s physical size, while NST can be more genetically defined. Thus, the genetic basis of cold adaptation in humans is more related to NST than it is to ST.

The uncoupling protein (UCP) gene family is one of the candidates contributing to humans’ cold adaptation. The UCPs are members of the MACP (mitochondrial anion carrier protein) family and play a role in generating heat when they separate oxidative phosphorylation from ATP synthesis. Humans have eight paralogous genes on the genome. Among them, three *UCP* genes (*UCP1, UCP2*, and *UCP3*) are related to thermogenesis with tissue specificity^3, 4^. Of those three genes, *UCP1* is expressed highly and especially in BAT, so that thermogenesis in adult humans is more active in winter^5^. Its expression level increases by repeating cold stimuli in lab experiments^6^, and under cold circumstances with hypobaric hypoxia in rats^7^. Therefore, the *UCP1* is considered major contributors to NST^2, 3^.

A polymorphism, located on the upstream of *UCP1*, alters its gene expression level^8^: the A allele at nucleotide position −3826 (rs1800592) is associated with a higher resting metabolic rate, a slower rate of weight gain^9^, and a higher postprandial thermogenesis in children^10^, compared with that of the G allele. The relationship between polymorphisms in the *UCP1* and the variation in physiological reaction has, therefore, been studied for its association with the obesity risk^11, 12^; however, in greater perspective, the polymorphism of the *UCP1* upstream is related to the basal metabolic rate^13^. Recent GWAS (genome-wide association studies) using northern Europeans and Siberians reported that some genes including the *UCP1* experienced natural selection against cold climate^14, 15^. However, to our knowledge, there is no direct experimental evidence that has definitively shown the difference in physiological response against the cold environment between the variants of *UCP1* in humans. In the present study, we examined the difference among the *UCP1* genotyping results in experiments on 47 subjects. They were exposed to cold (16 °C) in an artificial climate chamber to precipitate only NST, and their single nucleotide polymorphisms (SNPs) across 40 kb covering the *UCP1* region were genotyped. We show that the physiological responses to experimentally cold environments are significantly different among the *UCP1* genotypes.

## Results

### Physiological responses in our subjects

Table 1 summarizes physiological data at temperatures 16 °C and 28 °C. The subjects’ rectal (i.e., core) temperatures decreased approximately 0.5 °C at 16 °C, but there were no significant differences in the physiological measurements at 28 °C. The subjects’ skin (i.e., surface) temperatures were already lower at 16 °C than those at 28 °C at 0 min, and gradually decreased 1.4 °C at 16 °C after 90 min because of vasoconstriction, but there were no significant differences in the physiological measurements at 28 °C. Therefore, subjects at 16 °C were exposed to enough cold, but not overly severe because the decrease in core temperature was only 0.5 °C. On the other hand, subjects at 28 °C stayed in thermoneutral environment because of no change in body temperature. Noteworthy, oxygen consumption (VO_2_) was increased moderately in 60 min to 90 min at 16 °C without shivering. Thus, at 16 °C vasoconstriction of the subjects occurred to maintain core temperature; and additionally increasing VO_2_ could indicate thermogenesis occurring, not caused by ST but by NST. No physical characteristics of the subjects were associated with these physiological changes (Supplementary Table S1).

**Table 1:** Time series variations of physiological data under 16 and 28 degrees Celsius.

Measurements	Room Temperature	Time Course			
		0 min	30 min	60 min	90 min
VO_2_ (ml/kg/min)	16 °C	3.84 ± 0.72	3.83 ± 0.72 (3.91 ± 0.87)	3.98 ± 0.82	4.29 ± 0.90 (4.40 ± 1.17)
	28 °C	4.02 ± 0.86	3.57 ± 0.81	3.67 ± 0.64	3.72 ± 0.66
Rectal Temperature (°C)	16 °C	37.12 ± 0.29	36.93 ± 0.27	36.76 ± 0.28	36.61 ± 0.34
	28 °C	36.97 ± 0.26	36.9 ± 0.24	36.85 ± 0.24	36.93 ± 0.24
Skin Temperature (°C)	16 °C	30.29 ± 0.97	30.01 ± 0.99	29.86 ± 0.96	28.87 ± 0.98
	28 °C	34.13 ± 0.59	34.37 ± 0.57	34.48 ± 0.53	34.26 ± 0.47

### Difference of the oxygen consumption attributed to NST among *UCP1* genotypes and haplotypes

**Figure 1:**
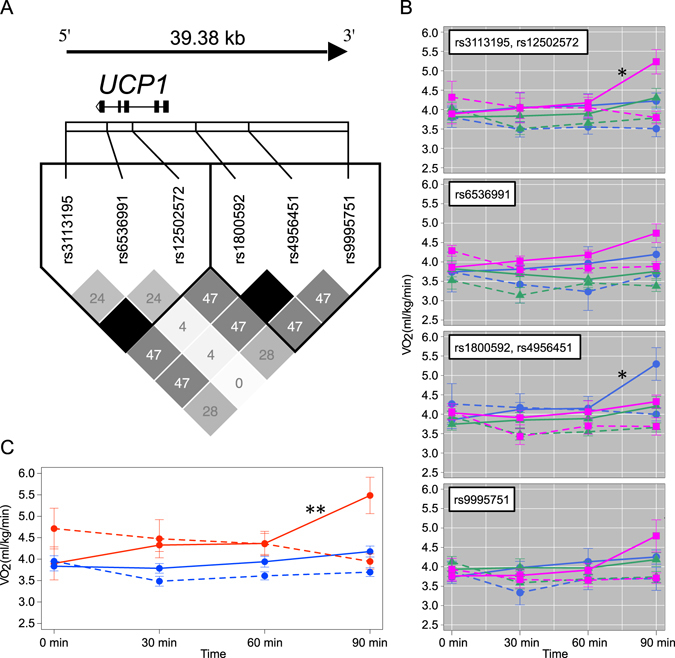
Physiological differences among UCP1 genotypes. (**a**) The physical positions and LD structure of UCP1 SNPs genotyped in this study. A pairwise *r* ^2^ value is shown in each square. Darker gradient color indicates higher *r* ^2^ values, and black indicates an *r* ^2^ of 1. The haplotype block defined by the Gabriel *et al*. method^31^ is represented by the enclosure of the black line. (**b**) Time series variations of VO_2_ (+/− s.e.m.) in each SNP and that genotype. The solid and dashed lines show the changes under 16 and 28 degrees Celsius, respectively. The colors of the lines represent each genotype (blue: a homozygote of an ancestral allele; magenta: a homozygote of a derived allele; green: heterozygote of ancestral/derived alleles). (**c**) Time series variations of VO_2_ (+/− s.e.m.) between haplotypes. The solid and dashed lines show the changes under 16 and 28 degrees Celsius, respectively. The colors of the lines represent each genotype (red: a homozygote of GnGTAn haplotype; blue: a homozygote and heterozygote of other haplotypes). The asterisks indicate a significant increase compared with other genotypes (*P < 0.05; **P < 0.01).

To examine the relationship between the physiological changes and the genetic variations on the *UCP1* gene region (almost 40 kb), we genotyped 6 SNPs of 47 subjects after the physiological experiments (Table 2). Significant deviations of the Hardy-Weinberg equilibrium (HWE) were not observed in any of the loci, and the haplotype frequencies on our subjects were almost consistent with those of JPT in the 1000 genome database (*P* = 0.148 by Fisher’s Exact Test, and see also Supplementary Fig. S1). Additionally, the *F*_st_ value indicated no genetic differentiation between our subjects and JPT (*F*_st_ = 0.00297). These statistics indicated that the subjects examined in the present study did not constitute a biased population. These results agree with previous studies using the whole genome SNP data^16, 17^, showing no significant population structure within the people from Mainland Japan including the Kyushu Island. We found the linkage disequilibrium (LD) values between rs3113195 and rs1250572, and between rs1800592 and rs4956451 were, each and all, “1,” indicating perfect links (Fig. 1a). For the subsequent analyses, therefore, we used 4 of 6 SNPs omitting 1 of the 2 that were linked. Classifying the 47 subjects into 3 genotypes (homozygote of ancestral alleles: AA, homozygote of derived alleles: DD, and heterozygote of ancestral/derived alleles: AD), we tested if the 4 SNPs (rs3113195, rs6536991, rs1800592, and rs9995751) are associated with the change of VO_2_ that was a measurement indicating the amount of thermogenesis. We focused on an increase of VO_2_ values from 60 min to 90 min because the clear increase after 60 minutes was reported in previous studies^18, 19^. The differences of the VO_2_ values were calculated by subtracting the values at 60 min from those at 90 min. Comparing the differences of the VO_2_ by each genotype, we found significant differences in the change of VO_2_ among the genotypes, rs3113195 and rs1800592 (Fig. 1b). VO_2_ was significantly increased at 60 min to 90 min in the homozygote of ancestral allele at rs3113195 (DD vs. AD: *P* = 5.9 × 10^−4^; DD vs. AA: *P* = 1.1 × 10^−3^, significance levels adjusted by the Holm’s method were 4.2 × 10^−3^ and 4.6 × 10^−3^, respectively). Similarly, VO_2_ was significantly increased at 60 min to 90 min in the homozygote of derived alleles at rs1800592 (AA vs. AD: *P* = 2.4 × 10^−3^; AA vs. DD: *P* = 8.1 × 10^−3^, significance levels adjusted by the Holm’s method were 5.0 × 10^−3^ and 5.6 × 10^−3^, respectively). The homozygote of these alleles showed significantly higher thermogenesis than the other states of genotypes. Considering the physiological responses with each genetic state, we would suggest that the haplotype GnGTAn (“n” represents an arbitrary nucleotide) has the highest thermogenesis among all the haplotypes. We found 4 subjects had the homozygote, GnGTAn. When testing the difference of the VO_2_ values (the subtracted the value at 60 min from that at 90 min) between the haplotype GnGTAn and the other haplotypes, the value of the haplotype GnGTAn was significantly higher than those of the others (Fig. 1c, the mean in GnGTAn: 1.120; the mean in the others: 0.248, *P* = 5.6 × 10^−3^ by Welch’s *t*-test), indicating that VO_2_ increased at 60 min to 90 min. Regarding to the other physiological responses, we could not detect obvious differences among the *UCP1* genotypes. The results indicated that the haplotype GnGTAn on the *UCP1* gene region could more effectively cause thermogenesis under cold conditions than could the other haplotypes.

**Table 2:** Summary statistics of 6 SNPs genotyped in this study.

SNP	N	Allele type		Number of each genotype			Ancestral Allele freq.	H_obs_	H_exp_	HWE (*P* value)
		Ancestral	Derived	AA	AD	DD				
rs3113195	47	A	G	11	30	6	0.447	0.638	0.494	0.100
rs6536991	47	C	T	4	14	29	0.234	0.298	0.359	0.395
rs12502572	47	A	G	11	30	6	0.447	0.638	0.494	0.100
rs1800592	47	T	C	7	27	13	0.436	0.574	0.492	0.433
rs4956451	47	A	G	7	27	13	0.436	0.574	0.492	0.433
rs9995751	47	C	T	8	23	16	0.415	0.489	0.486	1.000

AA, DD, and AD represent a homozygote of an ancestral allele, a homozygote of a derived allele, and a heterozygote of ancestral/derived alleles, respectively. H_obs_ and H_exp_ indicate an observed and expected heterozygosity, respectively. HWE indicates the Hardy-Weinberg equilibrium, and those values are the probabilities that did not deviate from the HWE.

**Figure 2:**
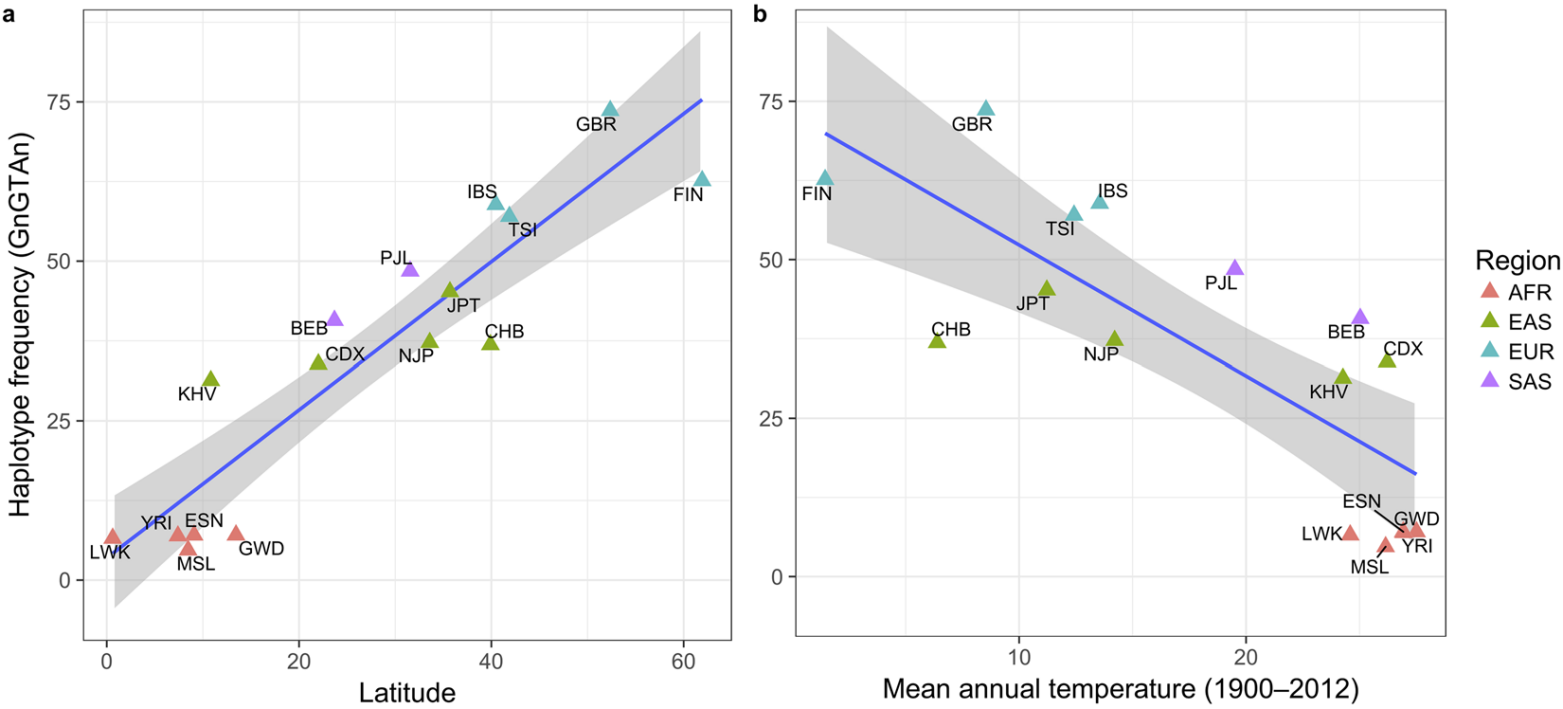
Correlation between the haplotype showing the highest efficiency thermogenesis and the latitudes (**a**) and mean annual temperatures (**b**) of global human populations deposited in the 1000 Genomes Project database. Blue line and grey band represent a regression line and its 95% confidence interval. Each color of triangle indicates the geographical region of populations. Dashed line represents a regression line, and orange triangle indicates the frequency and latitude of our subjects. The abbreviations used are the following: African (AFR): ESN (Esan in Nigeria), GWD (Gambian in Western Divisions in the Gambia), LWK (Luhya in Webuye, Kenya), MSL (Mende in Sierra Leone), YRI (Yoruba in Ibadan, Nigeria); European (EUR): FIN (Finnish in Finland), GBR (British in England and Scotland), IBS(Iberian Population in Spain), TSI (Toscani in Italia); East Asian (EAS): CDX (Chinese Dai in Xishuangbanna, China), CHB (Han Chinese in Bejing, China), JPT (Japanese in Tokyo, Japan), NJP (Japanese in this study), KHV (Kinh in Ho Chi Minh City, Vietnam); South Asian (SAS): BEB (Bengali from Bangladesh), PJL (Punjabi from Lahore, Pakistan).

Previous studies suggested that positive selection had acted on the *UCP1* gene regarding cold adaptation^15, 20^. We looked into the haplotype frequency of GnGTAn in the 1000 Genomes Project database, and examined the relationship between the frequency of the haplotype and the latitudes or mean annual temperature in the geographically distinct populations. We found that the GnGTAn frequency was very well correlated with latitudes and temperature in which the various populations lived (Fig. 2; Spearman’s rank correlation coefficients rho = 0.938 for latitude, rho = −0.756 for temperature. Note that the *p*-values for the correlations were omitted because the haplotype frequencies in worldwide populations could be affected by human migration history and were not independent statistically). These correlations were stronger than that of rs1800592 alone (Supplementary Table S2). Namely, the GnGTAn frequency was much higher in the high-latitude/low-temperature populations.

## Discussion

In this study, we provide direct association that the *UCP1* genotype affects NST generated in the subjects in the climatic chamber, kept at 16 °C which less frequently causes ST of skeletal muscles. We expected body temperatures would increase after increasing VO_2_ because thermogenesis maintains or increases body temperature. There were, however, no significant differences between the rectal and skin temperatures. The effect of NST on body temperature took longer than did that of ST. And then skin temperatures around BAT, which expressed much *UCP1* might have increased, but this did not prove these changes. Given that there were no differences of VO_2_ between the *UCP1* genotypes earlier during the cold exposure (i.e. the significant difference of VO_2_ appeared from 60 min to 90 min), this could be caused by the gene expression change of *UCP1*, rather than by the protein function change. Previous physiological studies showed that VO_2_ were clearly increased after at least 60 minutes under moderate cold exposure^18, 19^. A previous genetic study suggested that the SNP, rs1800592, on the promoter region of *UCP1* is statistically associated with the basal metabolism that is related to thermogenesis^13^, and that the genomic region around rs1800592 regulated the *UCP1* gene expression in humans^8^. Taken together with these studies, the observed time lag of increasing VO_2_ in rs3113195 and rs1800592 suggests that the causal mutations that change the gene expression could exist around those SNPs in non-coding regions.

This study also showed physiological association that the *UCP1* polymorphisms had driven a habitat range expansion in humans from the African to the Eurasian continents. The frequency of the haplotype GnGTAn which was composed of 6 SNPs was 37.2% in the subjects in the present study, which was higher in the European populations (ave. 63.0%), and lower in African populations close to the equator (ave. 6.5%, Supplementary Fig. S1). Typically, the temperature is lower at high latitudes. Because, in humans, the BAT that expresses the *UCP1* gene is richer in infants than that in adults, infants having GnGTAn more likely survive in cold circumstances. A previous study reported the *UCP1* gene region showed the signal acting on natural selection in northern European based on the integrated haplotype score and long-range haplotype statistics, the *UCP1* gene is a plausible candidate for adaptation to cold environments^15^. Furthermore, a genome wide scan of northern Inuits indicated a brown adipocyte-induced gene, *TBX15* ^21^, was under the selective pressure, which might have resulted in an increased number of cells capable of expressing *UCP1* in the Inuit population^22^. While the thermogenesis changes in BAT, which were related to a *UCP1* cascade, played an important role in humans’ cold adaptation, they might also contribute to an increase in basal metabolism for modern dietary habits containing more lipids, and reduce the risk of lifestyle diseases such as obesity in the present day under less cold stress than that in the past^23^. The present study supports this scenario physiologically, and builds consistency into the relationships between cold adaptation and the risk of obesity, as it is related to *UCP1*.

In the present study, we found 2 SNPs, rs3113195 and rs1250572, that showed NST differences, which were evaluated by VO_2_ among genotypes under cold exposure, in addition to rs1800592 that had previously been reported. Although we are unsure if these SNPs directly affect the gene expression of *UCP1* or if the other mutation(s) between the SNPs affect the gene’s expression, future studies are warranted to examine the region in detail, including the promoter and enhancer effects. Interestingly, these SNPs did not link to the SNPs that are already known, but rather strongly linked to the neighbor gene, *ELMOD2*, which might play an important role in antivirals, in the 1000 Genomes Project East Asia (JPT) (Supplementary Fig. S3)^24^. The elevation in body temperature can improve the immune system^25^. The combination between the *UCP1* gene and the *ELMOD2* gene, in both of which the frequencies increased at the same time, could be an important piece of evidence suggesting that humans adapted to cold climates, as well as unknown viruses, throughout human history.

Lastly, our study still leaves two challenges but will be able to solve them in the near future: the elimination of possible influence of genes linking the *UCP1* gene in our subjects, and the confirmation of body temperature rise by NST in subjects with GnGTAn haplotype. For the former, we would increase the number of samples so that a genome-wide approach can be applied, and more clarify the causal relationship between *UCP1* and NST as eliminating the effect of linkage disequilibrium. For the latter, we could be able to observe an increase in body temperature due to NST by prolonging the observational time for the subjects. Nonetheless, this study must be highly significant from the perspective of having shown experimentally the association between the genotype (haplotype) of *UCP1* and the change in oxygen consumption caused by NST using healthy subjects.

## Methods

### Subjects and physiological data collection

A total of 47 volunteers (undergraduate and graduate students in Kyushu University, Fukuoka, Japan; all males, 20 to 24 years old) who exhibited no clinical problems participated as subjects in the present study. The Kyushu Island is one of large four islands of Mainland Japan. Kyushu University is in the Fukuoka city in the northern part of the Kyushu Island, and the students of Kyushu University are mainly from the local area. The Kyushu University Institutional Review Board for Human Genome/Gene Research and the Kitasato University Research Ethics Committee approved this study protocol and all procedures were carried out in accordance with the approved guidelines. After having the experimental procedure described to the subjects, written informed consent was obtained from all of them.

The mean values and standard deviations of the physical characteristics of these 47 subjects were: height, 1.72 ± 0.05 m; weight, 61.08 ± 12.27 kg; body mass index (BMI), 20.76 ± 3.96 and body surface area (BSA), 1.66 ± 0.15 m^2^. The participants abstained from food and drink for more than 2 hours prior to entering the controlled climate chamber. The experiment was conducted with the subjects wearing only T-shirts and shorts and sitting quietly in chairs.

Measurement sensors (temperature sensors and gas analyzers) were attached to the participants in an environment with a temperature of 28 °C for 30 min prior to the experiment, and subsequently the participants entered the artificial climate chamber kept at the temperatures according to the experimental protocol. The ambient temperatures during the experiment were constant, either at 28 °C or 16 °C according to the conditions of the experimental protocol. Rectal temperature probes were inserted to the depth of 13 cm beyond the anal sphincter. Skin temperature sensors were attached with surgical tape to measurement sites on the forehead, abdomen, forearm, hand, thigh, leg, and foot. Mean skin temperature was calculated using the seven-point method of Hardy-DuBois see details in^﻿﻿19, 26^. Oxygen saturation (SpO_2_) measurements were made at 1-min intervals using the Pulse Oximeter Radical-7^TM^ (Massimo Corporation, Tokyo, Japan) and at each time point (0, 30, 60, and 90 min). Rectal temperature, skin temperature and SpO_2_ were measured consecutively throughout the experiments. Collection of exhaled gas (Douglas bag method) was carried out 4 times with steady-state atmospheric pressure at each time point (0, 30, 60, and 90 min). The VO_2_ was measured with a respiratory gas analyzer (AE-300S, Minato Medical Science, Osaka, Japan). VO_2_ indicates human energy expenditure via expiration gas, which corresponds to ST and NST in a resting state. SpO_2_ indicates a ratio of HbO_2_ (oxygenated hemoglobin) to total arterial hemoglobin estimated by the infrared light of the pulse oximeter. BAT is activated under hypobaric hypoxia^7^. Therefore, after cold exposure, air pressure was moderately decreased at 30 min and 60 min.

### DNA extraction and TaqMan SNP typing

Saliva samples were collected from the subjects. Total DNA was extracted using a Saliva DNA Isolation Kit (Norgen Biotek Corporation, Thorold, ON, Canada) according to the manufacture’s protocol. We selected 6 SNPs (rs3113195, rs6536991, rs12502572, rs1800592, rs4956451, and rs9995751), the allele frequencies of which are over 30% in the HapMap JPT except rs6536991 (20%), as covering the whole *UCP1* gene region at intervals of approximately 3000–10000 bp. The SNPs were genotyped by TaqMan SNP genotyping assays (Applied Biosystems, Tokyo, Japan) with the LightCycler 480 Probes Master (Roche Diagnostics, Tokyo, Japan) on the LightCycler 480 System II (Roche Diagnostics, Tokyo, Japan) according to the manufacture’s protocol. The HapMap database version we used was “HapMap Data Rel 28 PhaseII + III, August10, on NCBI B36 assembly, dbSNP b126.

### Statistical analysis

Outliers of five physiological measurements (VO_2_, SpO_2_, heart rate, rectal temperature and skin temperature) were detected by the Smirnov-Grubbs test for excluding abnormal values. *P* values less than 0.00125 were considered as statistically significant according to the Bonferroni’s correction (0.05 divided by 40 tests, 5 physiological measurements times 4 time-points times 2 chamber temperatures). The ancestral alleles for all the SNP sites were inferred by comparing them to chimpanzee, gorilla, and orangutan reference sequences. For all the SNPs, we examined whether the HWE was significantly rejected at *P* < 0.05 by the *χ* ^2^ test. Phased haplotypes were estimated on the basis of the Bayesian statistical method using PHASE v.2.1^27, 28^ with default setting in a DnaSP version 5.10.1 program^29^. Pairwise LD parameters, *r* ^2^, among the 6 SNP sites were estimated using the software, Haploview^30^, and haplotype blocks were defined by Gabriel *et al*.’s method^31^.

To test if the 4 SNPs are associated with the NST statistically, we focused on an increase of VO_2_ values from 60 min to 90 min because previous physiological studies showed the clear increase of VO_2_ were observed after 60 minutes under moderate cold exposure^18, 19^. The differences of the VO_2_ values were calculated by subtracting the values at 60 min from the values at 90 min. The significant differences of VO_2_ values among 3 genotypes (homozygote of ancestral alleles: AA, homozygote of derived alleles: DD, and heterozygote of ancestral/derived alleles: AD) for 4 SNPs were detected by the Welch’s t test (i.e., a total of 12 tests was repeated.), and the significance level was adjusted by Holm’s method. Subsequent statistical tests for the difference of the VO_2_ between the haplotypes were performed by Welch’s t test. To examine the association between other four physiological responses and *UCP1* genotypes, we plotted relationships between the physiological measurements and the genotypes of each SNP on *UCP1*. The associations of height, weight, BMI and BSA with the differences of VO_2_ were assessed by multiple regression analyses.

To view a relationship between UCP1 haplotypes and environmental variables across worldwide populations, we selected 15 populations (Africa: ESN, GWD, LWK, MSL, YRI; Europe: FIN, GBR, IBS, TSI; East and South Asia: CDX, CHB, JPT, KHV, BEB, PJL) (see the abbreviations in Fig. 2 **)** from the 1000 Genomes Project database, where the latitude and temperature of their habitats could be identified using Google Map and Climate Change Knowledge Portal by the world bank group. In temperature data, we used the mean annual temperature during 1900 to 2012 of individual countries for each population who mainly belongs to, excepting YRI, CDX and NJP. The mean annual temperature of Lagos (local city in Nigeria) was applied for YRI, Thailand for CDX, Fukuoka (local city in Japan) for NJP. We calculated the frequencies of the cold adaptive haplotype, GnGTAn, and the Spearman’s rank correlation coefficients between the GnGTAn frequencies and the latitudes/mean annual temperatures. Lastly, we calculated the LD values among 13 SNPs including the 6 SNPs mentioned above and an additional 7 SNPs covering the neighboring gene, *ELMOD2*, using 104 JPT samples in the 1000 Genomes Project database: we chose the 7 SNPs with more than 30% at the allele frequencies again in the HapMap JPT except rs11100662. These statistical analyses were performed using the R 3.1.1 software.

## Electronic supplementary material

Supplementary Materials
