# Supplementary material for: Experimental evidence reveals the UCP1 genotype changes the oxygen consumption attributed to non-shivering thermogenesis in humans
Source: Sci Rep. 2017 Jul 17;7:5570. doi: 10.1038/s41598-017-05766-3 (PMC5514118; doi:10.1038/s41598-017-05766-3)
Supplement: Supplementary file 1 — Supplementary Materials [file 41598_2017_5766_MOESM1_ESM.pdf]

Supplementary Materials

Experimental evidence reveals the *UCP1* genotype changes the oxygen consumption attributed to non-shivering thermogenesis in humans

Takayuki Nishimura[1,4], Takafumi Katsumura[2,4], Midori Motoi[1], Hiroki Oota[2*], Shigeki Watanuki[3*]

[1]Department of Biomedical Sciences, Nagasaki University, 1-12-4 Sakamoto, Nagasaki, 852-8523, Japan.

[2]Department of Anatomy, Kitasato University School of Medicine, 1-15-1 Kitasato, Minami-ku, Sagamihara, Kanagawa, 252-0374, Japan.

[3]Deparatment of Human Science, Kyushu University, 4-9-1 Shiobaru, Minami-ku, Fukuoka, 815-8540, Japan.

[4]These authors contributed equally to this work.

*Correspondence should be addressed to H.O. (hiroki_oota@med.kitasato-u.ac.jp) or S.W. (watanuki@design.kyushu-u.ac.jp).

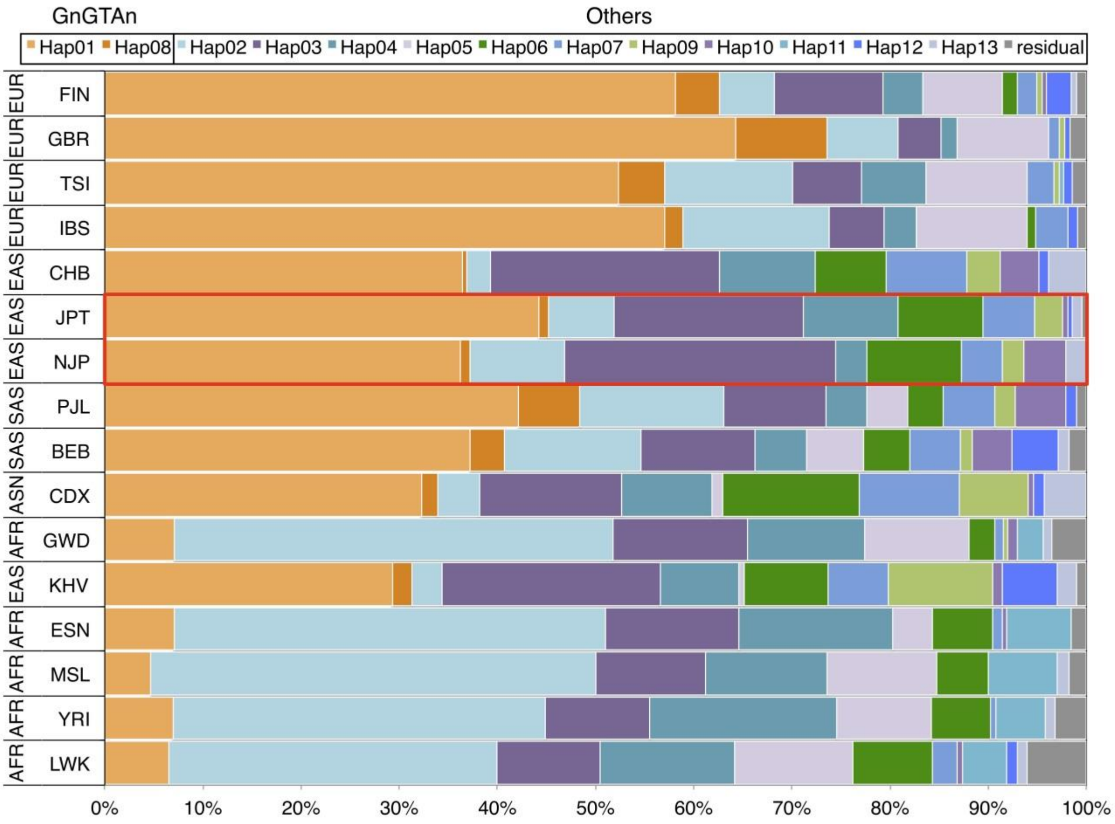

Supplementary Fig. S1. Haplotype frequency of *UCP1* based on 6 SNPs genotyped in this study. Red frame showed a comparison of the haplotype frequency between JPT and NJP, which no difference in the frequency was observed (P = 0.148, Fisher's Exact Test). These populations in the graph are arranged in descending order of latitude. The abbreviations used are described in Fig. 2.

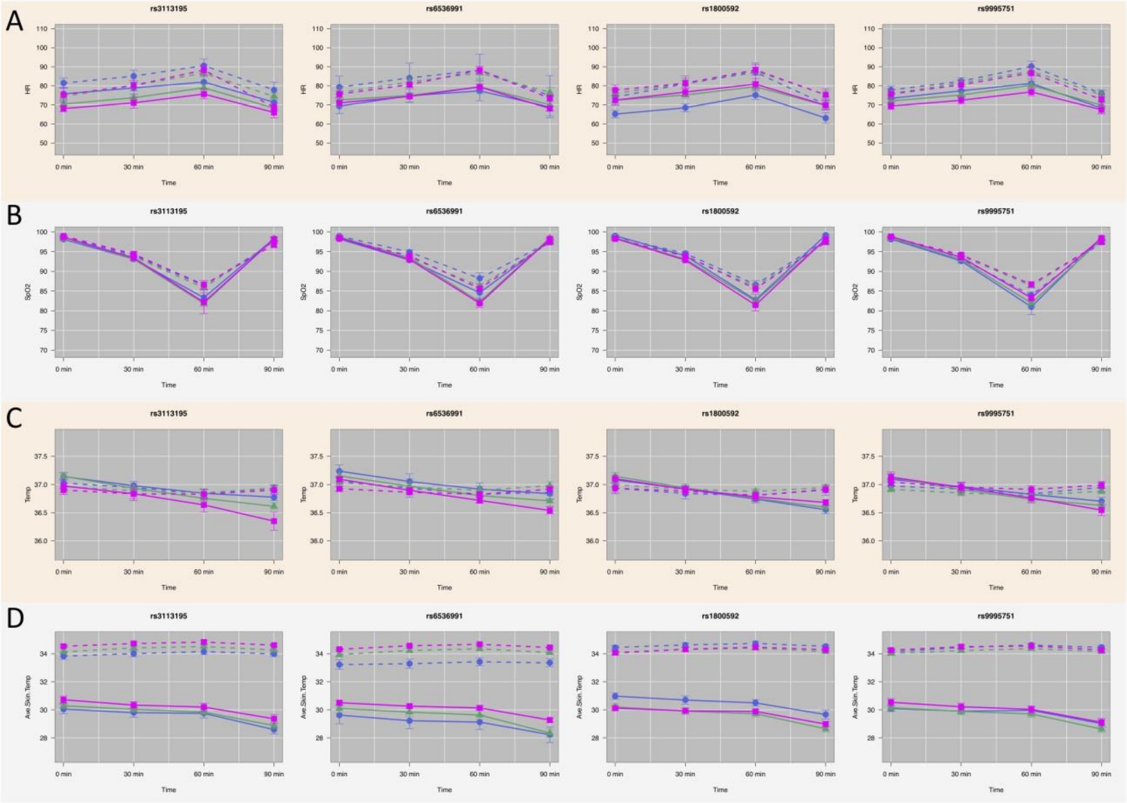

Supplementary Fig. S2. Time series variations of physiological data in each SNP and genotype (A: Heart rate; B: SpO_2; C: Rectal temperature; D: Skin temperature). The solid and dashed lines show the changes under 16 and 28 degrees Celsius, respectively. The colors of the lines represent each genotype (blue: a homozygote of an ancestral allele; magenta: a homozygote of a derived allele; green: heterozygote of ancestral/derived alleles).

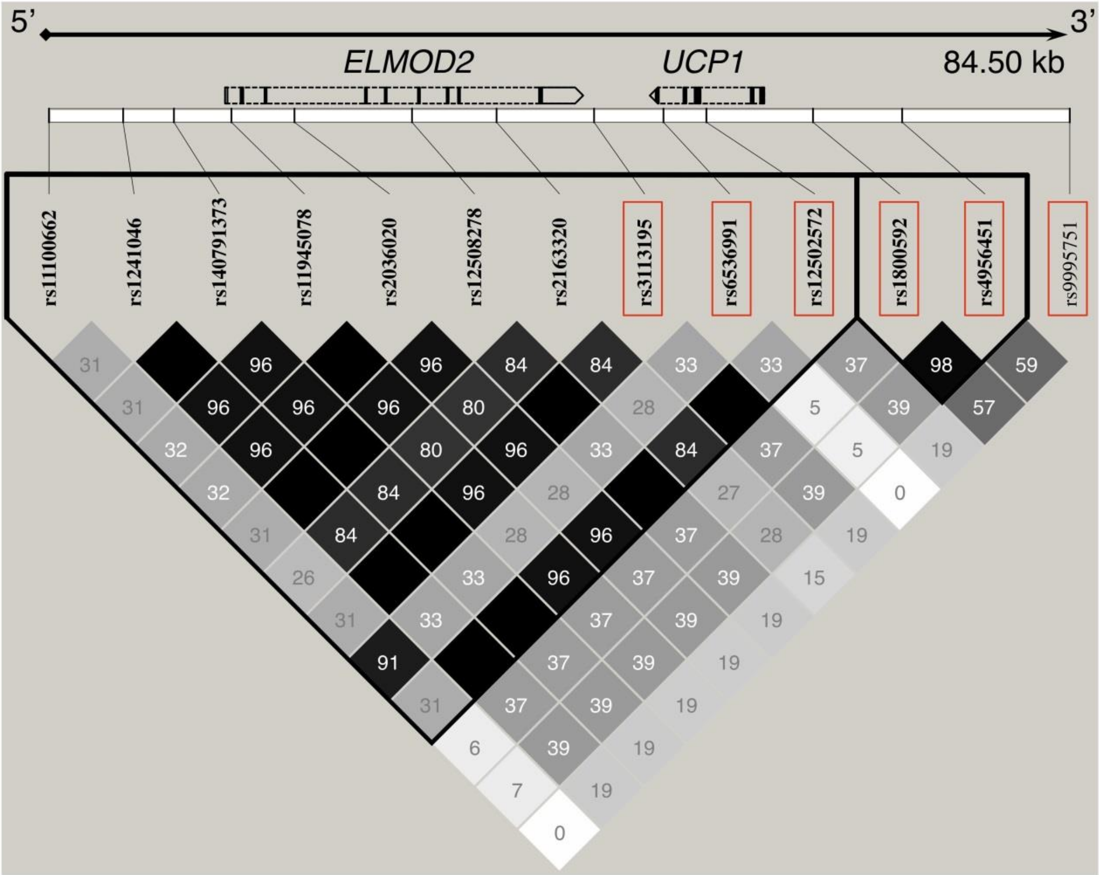

Supplementary Fig. S3. LD structure around the *UCP1* and *ELMOD2* genes. A pairwise r^2 value is shown in each square. Darker gradient color indicates higher r^2 values, and black indicates an r^2 of 1. The haplotype block defined by the Gabriel et al. method[31] is represented by the enclosure of the black line. The red frame indicates the SNP genotyped in this study.

Supplementary Table S1. The results of a multiple regression analysis to examine the association of BM, BMI and BSA with Δ VO_2 (90min-60min). BM: Basal metabolic rate; BMI: Body mass index; BSA: Body surface area.

	Response variable		
	Δ VO_2 (90min-60min)		
Explanatory variables	Coefficients	S.E.	*p* value
(Intercept)	0.511	0.156	0.002
BM	-0.114	0.196	0.563
BMI	-0.400	0.289	0.174
BSA	0.274	0.275	0.325

Supplementary Table S2. Comparisons of Spearman rank correlation coefficients in this study and Hancock et al. (2011).

		Spearman's rho	
Haplotype or SNP	Database	Latitude	Mean annual temperature
GnGTAn	1000 Genomes	0.94	-0.76
rs1800592	1000 Genomes	0.81	-0.72
rs1800592	HDGP	0.62	-0.41
